# Comprehensive analysis of TCGA data reveals correlation between DNA methylation and alternative splicing

**DOI:** 10.1186/s12864-022-08992-w

**Published:** 2022-11-18

**Authors:** Shuting Lin, Soojin Yi, Peng Qiu

**Affiliations:** 1 School of Biological Sciences, Georgia Institute of Technology, Atlanta, USA; 2 Ecology, Evolution, Marine Biology, University of California, Santa Barbara, Santa Barbara, USA; 3 Department of Biomedical Engineering, Georgia Institute of Technology and Emory University, Atlanta, USA

**Keywords:** DNA methylation, Alternative splicing, Exon, Correlation analysis, Survival analysis

## Abstract

The effect of DNA methylation on the regulation of gene expression has been extensively discussed in the literature. However, the potential association between DNA methylation and alternative splicing is not understood well. In this study, we integrated multiple omics data types from The Cancer Genome Atlas (TCGA) and systematically examined the relationship between DNA methylation and alternative splicing. Using the methylation data and exon expression data, we identified many CpG sites significantly associated with exon expression in various types of cancers. We further observed that the direction and strength of significant CpG-exon correlation tended to be consistent across different cancer contexts, indicating that some CpG-exon correlation patterns reflect fundamental biological mechanisms that transcend tissue- and cancer- types. We also discovered that CpG sites correlated with exon expressions were more likely to be associated with patient survival outcomes compared to CpG sites that did not correlate with exon expressions. Furthermore, we found that CpG sites were more strongly correlated with exon expression than expression of isoforms harboring the corresponding exons. This observation suggests that a major effect of CpG methylation on alternative splicing may be related to the inclusion or exclusion of exons, which subsequently impacts the relative usage of various isoforms. Overall, our study revealed correlation patterns between DNA methylation and alternative splicing, which provides new insights into the role of methylation in the transcriptional process.

**Supplementary Information:**

The online version contains supplementary material available at 10.1186/s12864-022-08992-w.

## Background

DNA methylation is one of the most extensively studied epigenetic mechanisms and is essential for normal development and many key biological processes, including embryogenesis, genome imprinting, and regulation of gene transcription [1–3]. Although DNA methylation is known to be perturbed in various cancers, its role in tumorigenesis is not fully understood [4], motivating numerous studies to explore the mechanism of DNA methylation in human cancers  [5, 6]. A main question in this regard is the role of CpG methylation and gene expression. Many studies have characterized DNA methylation as a silencer or activator of gene transcription because methylation of the promoter region often represses gene expression, while methylation of some CpG sites in the gene body is correlated with increase of transcriptional activities [2, 7–9]. However, emerging evidence shows that DNA methylation not only affects transcription but also regulates alternative splicing [10, 11].

Alternative splicing is known to have significant impact on cancer [12, 13]. Yet, few studies have explored the correlation between methylation and alternative splicing, specifically the regulatory mechanisms of DNA methylation on alternative splicing in cancer. We speculate that this could be due to methylation being an epigenetic event that occurs on DNA, while alternative splicing occurs during the processing of RNA molecules, and therefore the possibility of a direct relationship between DNA methylation and alternative splicing has received less attention than it should have. The lack of explorations of the possible role of DNA methylation in alternative splicing presents an opportunity for further research, as it could contribute toward new understanding of DNA methylation during transcriptional processes, especially in tumorigenesis.

With advances in high-throughput genomics, extensive cancer databases such as The Cancer Genome Atlas (TCGA) have become publicly available to examine the relationship between DNA methylation and alternative splicing. TCGA provides multiple types of molecular data on cancer patients, including mutation, copy number variation, DNA methylation, gene expression, miRNA expression and protein expression, across 33 cancer types and a total of $\sim$11,000 cancer patients. Using TCGA data resources, some in vitro studies in the literature have incorporated DNA methylation data and transcript level quantification data to explore the potential link between alternative splicing and DNA methylation. For example, one previous study has used association analysis to characterize the underlying relationship between cancer-specific alternative splicing and DNA methylation, and observed several different patterns of methylation-alternative splicing correlations [14]. Another study developed a splicing decision model to identify actionable methylation loci potentially affecting splicing events, which revealed that intragenic methylation status is important for splicing regulation [15]. However, most of the previous studies were limited to specific cancers, or focused exclusively on differentially methylated CpGs between tumor and normal samples. In this study, we aimed to comprehensively integrate and analyze different molecular data types in TCGA, including data on DNA methylation, exon expression, isoform expression, and gene expression, to unravel the correlation pattern between methylation and alternative splicing across multiple cancer types.

**Fig. 1:**
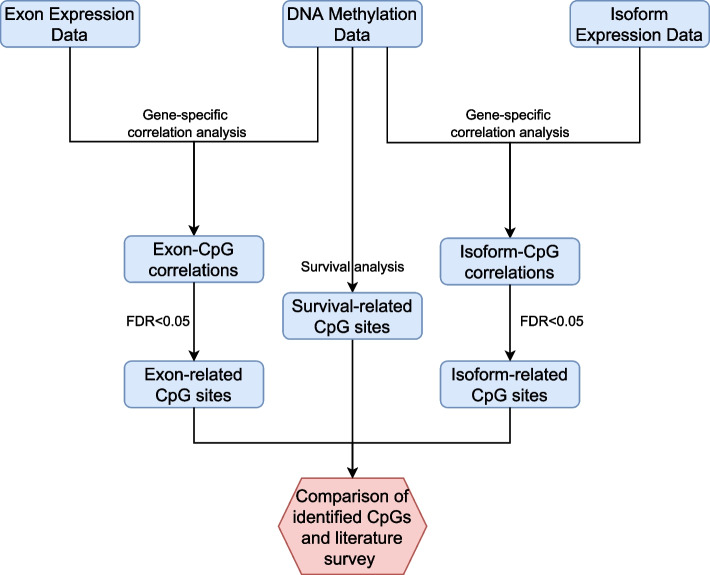
Flowchart of data analysis used in this study. First, we selected gene-specific samples that highly expressed the gene for each of the genes we analysed, and then we performed correlation analysis between DNA methylation and exon/isoform expression based on gene-specific samples. A threshold of FDR<0.05 was required to detect significant correlations. In addition, we performed survival analysis for each CpG sites and identified a list of CpG sites that are predictive of patients’ survival outcome. Finally, a literature survey was conducted to search for existing evidences of identified correlations

Here, we present a pan-cancer analysis to examine the relationship between DNA methylation and alternative splicing on an individual gene basis (Fig. 1). For one cancer and one gene, we selected patients with high expression of that specific gene, henceforth defined as “gene-specific patients”. We used the data on those patients to compute the correlations between DNA methylation levels of CpG sites associated with the gene and the expression data of exons of that gene. Thus, we identified genes whose DNA methylation was significantly correlated with alternative splicing in cancers. Next, we reviewed the literature to find evidence supporting the relationship between DNA methylation and alternative splicing in the identified genes. Furthermore, for each cancer, we stratified CpG sites into two classes (significant CpGs vs. non-significant CpGs), depending on whether the CpG sites were significantly associated with at least one of the exons of the corresponding gene. In order to evaluate whether there are differences in these two classes in terms of their ability to predict survival outcomes, we applied survival analysis to all the individual CpG sites and found CpG sites that correlate with exons are more likely to be correlated with survival, compared to the CpG sites that do not correlate with exons. In addition, we applied a correlation analysis between the CpG sites and isoforms for each gene in each cancer type. Interestingly, we observed that CpG sites are more strongly correlated with exon expressions than their correlations with isoform expressions. This observation indicates that CpG methylation may affect alternative splicing by regulating the inclusion or exclusion of exons, which subsequently impacts the expression and usage at the isoform level.

## Results

### Correlations between DNA methylation and alternative splicing

For each of the 33 cancer types in TCGA and each gene, we performed a correlation analysis of the DNA methylation data for each CpG site and the expression data of each exon of the corresponding gene, which involved with a total of 485,577 CpG sites and 239,322 exons in 20,880 genes. On average, one gene was associated with 22 CpG sites and had 9.65 exons, which amounted to examining a total of 134,299,062 CpG-exon correlations.

For each cancer type and each gene, patients belonging to the cancer type were stratified into a highly-expressed group and a lowly-expressed group by applying StepMiner [16] to binarize the gene expression data of the gene. Specifically, we sorted the expression data of the gene for all patients in all cancer types, and then fitted a step function to the sorted data that minimized the squared error between the sorted data and the fitted step function, which provided a global threshold to binarize the expression of the gene. Since this threshold was derived based on the data of all patients across all cancer types, it robustly defined high and low expression of the gene. Since we were interested in studying alternative splicing, we focused on patients in the highly-expressed group for the specific gene of interest in the cancer type. We then calculated the correlations between DNA methylation of each of the CpG sites and expression of each of the exons for the given gene based on those patients who highly expressed the gene in that cancer type. In addition, we required that correlation analysis can only be performed when there were at least 10 patients for a given gene and a given cancer type to avoid bias due to small sample size. After computing CpG-exon correlations for each gene in each cancer type, we identified significant CpG-exon correlations using a 5$\%$ false discovery rate (FDR) threshold. The correlation analysis in 33 cancer types led to 6,049,182 significant CpG-exon correlations associated with 280,838 unique CpG sites and 190,577 unique exons in 19,984 genes, which we called “significant CpG sites” and “significant exons”, respectively. Since the correlation analysis was performed for each cancer and each gene separately, it is possible that a CpG site may correlate with multiple exons in the corresponding gene, and their association may appear in different cancer contexts. We focused on CpG-exon correlations that were significant in multiple cancer types in this study, because these CpG sites may play important role in exon usage during transcriptional processes and carcinogenesis. A total of 36,470 CpG-exon correlations were significant in more than one cancer type, which we called multi-cancer CpG-exon correlations. The 30 strongest correlations we found are presented in Table 1. The entire table for the 36,470 CpG-exon correlation is available in the Supplementary File.1.

To better understand the association between DNA methylation and exon expression, we evaluated the consistency of correlation direction in each of the 36,470 CpG-exon pairs that showed significant correlation in multiple cancers. Specifically, for each of the 36,470 CpG-exon pairs, we extracted its corresponding CpG-exon correlation values that showed significance, and stratified these significant correlation values. This stratification defined two groups of values for a CpG-exon pair, a majority group consisting of more than half of the corresponding correlation values that shared the same correlation direction, and a minority group consisting of the remaining correlation values for the CpG-exon pair. After that, we computed a consistency score for the CpG-exon pair as the ratio between the number of correlations in the majority group and the total number of significant correlations for the CpG-exon pair. The consistency score ranged from 0.5 to 1, with 1 indicating a perfect consistency where all of the corresponding CpG-exon correlation values shared the same correlation direction, and 0.5 indicating poor consistency. Among the 36,470 CpG-exon pairs with significant correlations in multiple cancers, 78.71$\%$ showed a perfect consistency score of 1, and 84.52$\%$ showed a consistency score of >0.7. Therefore, when a CpG-exon pair showed significant correlations in multiple cancers, the directions of the correlations tended to be the same.

**Table 1:** Top 30 multi-cancer CpG-exon correlations across all the cancer types

CpG	Exon	Gene	Cancer	Correlation	Majority sign
cg23538703	chr3:118864997-118866434:+	RP11-484M3.5	breast; cervical	0.845	−
cg02425416	chr11:2170356-2170833:-	INS-IGF2	cholangiocarcinoma; liver	0.822	$+$
cg02425416	chr11:2170356-2170575:-	INS-IGF2	cholangiocarcinoma; liver	0.821	$+$
cg02425416	chr11:2168796-2169037:-	INS-IGF2	cholangiocarcinoma; liver	0.818	$+$
cg05777976	chr11:2170356-2170833:-	INS-IGF2	cholangiocarcinoma; liver	0.807	$+$
cg05777976	chr11:2170356-2170575:-	INS-IGF2	cholangiocarcinoma; liver	0.803	$+$
cg05777976	chr11:2168796-2169037:-	INS-IGF2	cholangiocarcinoma; liver	0.802	$+$
cg13167664	chr11:2170356-2170575:-	IGF2	cholangiocarcinoma; liver	0.792	$+$
cg04083712	chr7:99158156-99158318:+	GS1-259H13.10	colon; esophageal	0.786	−
cg13167664	chr11:2170356-2170833:-	IGF2	cholangiocarcinoma; liver	0.782	$+$
cg01814130	chr3:118906622-118906822:+	RP11-484M3.5	bladder; cervical	0.773	−
cg14458615	chr3:118864997-118866434:+	RP11-484M3.5	breast; cervical	0.766	−
cg12389423	chr3:118864997-118866434:+	RP11-484M3.5	breast; cervical	0.765	−
cg10066151	chr19:57874879-57875071:+	AC003002.4	cervical; brain	0.756	−
cg15744005	chr10:104632854-104632990:+	C10orf32-ASMT	breast; cervical	0.755	−
cg20675391	chr3:118864997-118866434:+	RP11-484M3.5	breast; cervical	0.751	−
cg15744005	chr10:104632205-104632355:+	C10orf32-ASMT	breast; cervical	0.750	−
cg27331871	chr11:2168796-2169037:-	INS-IGF2	cholangiocarcinoma; liver	0.749	$+$
cg05777976	chr11:2170356-2170833:-	IGF2	cholangiocarcinoma; liver	0.747	$+$
cg05777976	chr11:2170356-2170575:-	IGF2	cholangiocarcinoma; liver	0.746	$+$
cg02166532	chr11:2170356-2170575:-	IGF2	cholangiocarcinoma; liver	0.744	$+$
cg15744005	chr10:104629841-104629968:+	C10orf32-ASMT	breast; cervical	0.744	−
cg04334121	chr11:18257383-18257477:-	SAA4	cholangiocarcinoma; liver	0.743	−
cg16817891	chr11:2170356-2170575:-	INS-IGF2	cholangiocarcinoma; liver	0.743	$+$
cg04334121	chr11:18253942-18254080:-	SAA4	cholangiocarcinoma; liver	0.742	−
cg13167664	chr11:2168796-2169037:-	IGF2	cholangiocarcinoma; liver	0.741	$+$
cg16817891	chr11:2170356-2170833:-	INS-IGF2	cholangiocarcinoma; liver	0.740	$+$
cg27331871	chr11:2170356-2170833:-	INS-IGF2	cholangiocarcinoma; liver	0.740	$+$
cg20844262	chr20:62365997-62366176:+	ZGPAT	cholangiocarcinoma; liver	0.740	−

In Table 1, the multi-cancer CpG-exon pairs were sorted by the mean of absolute values of their significant correlations. We observed that these top CpG-exon pairs all showed relatively high correlations in various cancers. These top correlations are dominated by a few CpG positions and their associations with several genes. A literature search based on PubMed database was performed to further investigate the significance of these top CpG-exon correlations. Interestingly, we found supporting evidence for two of the top genes. One is insulin-like growth factor 2 (IGF2), an imprinted gene with the parental allele expressed and the maternal allele silenced [17]. Previous studies have reported that epigenetic features such as histone modifications and DNA methylation are associated with different levels of IGF2 transcription regulation such as alternative splicing [18]. The same CpG sites or nearby CpG sites also showed strong correlations with the exon expression of INS-IGF2, which is a read-through transcript composed of exons from proinsulin precursor (INS) and IGF2 [19]. In the literature, it was observed that hypomethylation of INS-IGF2 was correlated with increase of INS-IGF2 transcripts in four breast cancer cell lines  [20]. Our analysis, using an entirely different method, has identified these previously recognized relationships between DNA methylation and expression of these genes.

### CpG sites correlated with exons tended to be more predictive of survival outcomes

**Fig. 2:**
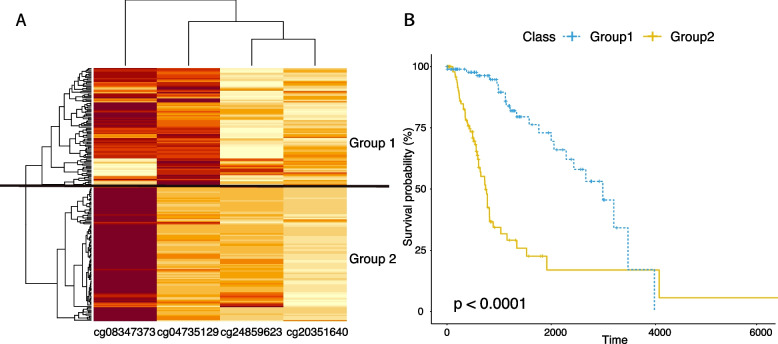
**A** Cluster heatmap of the methylation data of the CpG sites associated with CD302 in brain cancer. Patients were separated into two groups based on the clustering results. **B** Kaplan-Meyer survival curves of the two patient group stratified by the CpGs associated with CD302

To explore the differences between CpG sites with and without significant correlation with exons, we compared their ability to predict cancer survival outcomes. Specifically, for each cancer type and each CpG site, we focused on patients that highly expressed the gene associated with that CpG site, and performed survival analysis and log-rank test to examine whether methylation status of that CpG site was predictive of survival of patients who expressed the corresponding gene. This survival analysis in 33 cancer types identified 31,045 CpG sites that correlated with patient’s survival after a FDR correction for *p*-values (<0.05). Interestingly, when we compared the list of CpG sites correlated with the exons and the list of CpG sites associated with survival, we observed that 643 CpG sites were overlapped between the two lists, all of which were related to brain and breast tumors. According to the significance of survival differences, Table 2 shows the top CpG sites that correlated with both exon expression and survival outcomes. We performed a literature search on the genes associated with the overlapping CpG sites and found previous studies linked to them. For example, our results showed that multiple CpG sites associated with CD302 were predictive of survival in brain tumors (Fig. 2), which is consistent with another study showing that the expression level of CD302 was upregulated in brain regions and may be involved in biological processes such as development, differentiation, and immunological responses [21]. Specifically, we identified four CpGs associated with CD302 and are predictive of survival in brain cancer, which are cg08347373, cg04735129, cg24859623, and cg20351640. In order to examine their prognostic ability, we clustered 199 patients with brain cancer using the methylation data of the four survival-related CpGs (Fig. 2A), and observed two well-separated clusters. Next, we stratified patients into two groups, which are group1 and group2, according to the clustering results. Survival analysis was performed to examine the ability of the identified CpGs for stratifying patients with different prognoses (Fig. 2B).

**Table 2:** CpG sites that correlate with both exon expression and survival outcome

CpG	Gene	Cancer	*P*-value	FDR
cg20351640	CD302	brain	1.70E-08	0.000138852
cg03903831	RP11-307N16.6	brain	3.51E-08	0.000181116
cg27048140	ATP5J2-PTCD1	brain	8.93E-08	0.000289902
cg19857457	RPL17-C18orf32	breast	1.09E-08	0.000337161
cg03903831	SPATA13	brain	1.88E-07	0.000432463
cg05032848	RFFL	brain	2.45E-07	0.000497251
cg20477147	NPEPL1	brain	3.95E-07	0.000600647
cg24859623	CD302	brain	3.95E-07	0.000600647
cg09088496	RP11-307N16.6	brain	3.86E-07	0.000600647
cg04735129	CD302	brain	4.81E-07	0.000657035
cg08347373	CD302	brain	5.69E-07	0.000711062
cg08278937	SERINC4	brain	7.10E-07	0.000783884
cg16992627	TNXB	brain	8.01E-07	0.000830607
cg21870038	RFFL	brain	8.07E-07	0.000830823
cg13586610	TNXB	brain	1.07E-06	0.000969694
cg22158248	ALDH2	brain	1.23E-06	0.001040217
cg13799005	RP11-644F5.10	brain	1.25E-06	0.001049216
cg09088496	SPATA13	brain	1.97E-06	0.00123508
cg06017559	RP11-161M6.2	brain	2.34E-06	0.001332273
cg07204711	CDK3	brain	2.32E-06	0.001332273

*P-value and FDR are for log-rank test of CpG association with survival

**Fig. 3:**
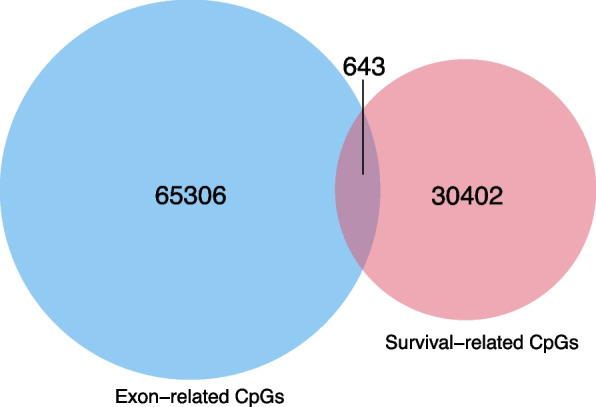
CpGs correlated with exons and survival. This Venn diagram shows the overlapping between the CpGs correlated with the exons and the CpGs that are predictive of survival

To assess whether the number of overlapping CpG sites that correlated with both exon and survival was statistically significant, we computed the expected number of overlaps in cases where the two relationships were independent. Since we analyzed data for 33 cancer types, the total number of CpG-survival relationships examined is the number of CpG sites (485,577) multiplied by 33. As mentioned above, we observed 31,045 significant CpG-survival relationships, which is roughly 0.19$\%$ of the total number of CpG-survival relationships examined. If the CpG-exon correlation was independent of the CpG-survival relationships, among the 65,949 CpG sites correlated to exons, only 124 are expected to be significantly correlated with survival. However, we observed 643 overlapping CpGs in both CpG-exon correlation analysis and survival analysis, which was 5.2 times greater than expected (Fig. 3). This observation showed that the CpG sites correlated with exons tended to be more correlated with cancer survival outcomes than expected, which could be an indication that CpG sites correlated with exons tend to have functional consequences on clinical outcomes [12, 13]. This result was consistent with those of previous studies regarding the ability of methylation-related alternative splicing events to predict survival outcomes in cancer [12–14]

### CpG sites are more strongly correlated with exons than their correlation with isoforms

We examined the relationship between DNA methylation and isoform expression. Similar to the analysis of CpG-exon correlation, for each cancer type and each gene, we first stratified patients into highly-expressed and lowly-expressed groups according to the binarized expression data for the gene. We only used patients in the highly-expressed group to calculate correlations between the methylation level of CpG sites associated with the gene and the expression data of isoforms of that gene. For a given cancer type, we computed the correlation for a given gene only if the number of patients in highly-expressed group exceeded 10. We applied FDR adjusted significance *p*-value < 0.05 as a threshold in each cancer type to identify significant CpG-isoform correlations in the 33 cancer types. A total of 1,929,993 CpG-isoform correlations exceeded the threshold, which involved 276,554 CpG sites, 131,735 isoforms, and 19,368 genes.

**Fig. 4:**
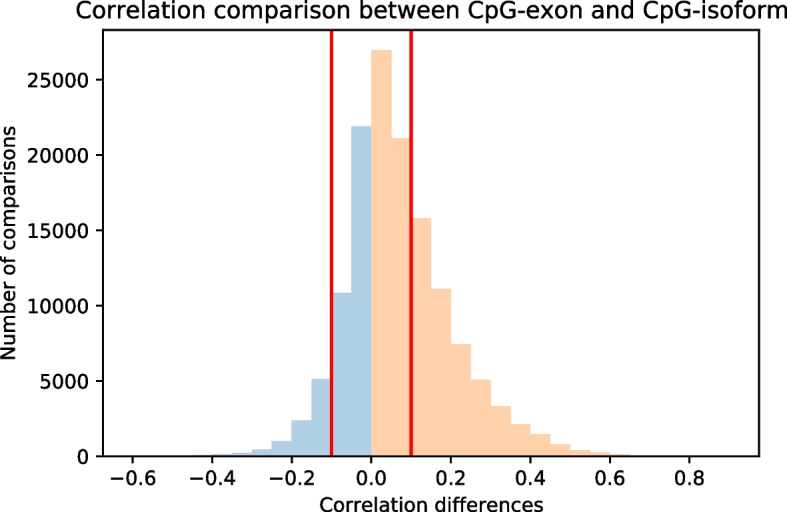
Comparison of correlation strength between CpG-exon and CpG-isoform. This histogram shows the differences between CpG-exon correlations and the corresponding strongest CpG-isoform correlations, which indicates that CpG-exon correlations tend to be stronger than the corresponding CpG-isoform correlations

We compared the strengths between CpG-exon correlations and CpG-isoform correlations, where the same CpG was significantly correlated with the expression of an exon as well as with an isoform containing the exon. Specifically, in each cancer type, for each of the CpG sites significantly correlated with an exon, we examined all the isoforms that correlated with the same CpG site and contained the exon. We then calculated the difference between the CpG-exon correlation and the corresponding CpG-isoform correlation to determine which of them was stronger. Since an exon can belong to multiple isoforms, it is possible that a CpG-exon correlation has multiple corresponding CpG-isoform correlations in the same cancer context, and these CpG-isoform correlations may have different strengths or even different directions. Therefore, for a given CpG-exon correlation and its corresponding CpG-isoform correlations, we selected the strongest CpG-isoform correlation with a direction consistent with the CpG-exon correlation. For example, if a CpG-exon pair was positively correlated, we selected the CpG-isoform that showed a positive correlation and had the largest correlation value. In contrast, if a CpG-exon pair was negatively correlated, we selected the negative CpG-isoform correlation with the smallest correlation value. We computed the differences between CpG-exon correlations and the corresponding strongest CpG-isoform correlations, and visualized the differences in the histogram in Fig. 4. It is interesting to observe that the histogram is skewed toward the positive side, with 70.20$\%$ of the differences greater than 0. When we set up $\pm 0.1$ thresholds for the correlation differences, 34.76$\%$ of the differences were $>0.1$, whereas only 6.81$\%$ of the differences were $<-0.1$, meaning that CpG-exon correlations tended to be stronger than the corresponding CpG-isoform correlations. This result may indicate that DNA methylation’s impact on the inclusion or exclusion of exons could subsequently affect isoform usage.

## Discussion

In this study, we examined the relationship between DNA methylation and alternative splicing by integrating multiple data types in TCGA. Our analyses successfully identified significant CpG-exon correlation patterns in various cancer contexts and explored the regulatory mechanism of CpG methylation on alternative splicing. Although most CpG-exon pairs showed a negative correlation in various cancer contexts, consistent with the concept of methylation-induced expression silencing, we also observed a substantial number of CpG-exon pairs exhibiting positive correlations. Remarkably, we showed that the majority of CpG-exon expression correlations had a consistent direction across multiple cancer types, indicating that the relations we have identified may share common molecular mechanisms across multiple cancers. Log-rank test was used to explore the connection between CpGs that correlated with exons and CpGs that correlated with survival outcomes. We showed that the CpG sites correlated with exon expression were enriched with CpG sites that correlated with survival outcomes, which may indicate that the CpGs correlated with exons have larger functional consequences than CpGs that do not correlate with exons. Therefore, the significant CpG-exon expression correlations we have identified in this study may provide useful candidates for functional studies in the future. Furthermore, we performed pairwise correlation analysis between CpG sites and isoforms for each cancer-gene combination, and compared the correlation strength between CpG-exon and CpG-isoform. Our analysis demonstrated stronger correlations between CpGs and exons compared to the correlations between CpGs and isoforms that contain the exons, indicating that CpG methylation may be associated with alternative splicing via regulating the inclusion or exclusion of exons, which subsequently impacts the relative usage of various isoforms.

This study is not without limitations. One limitation was the lack of independent validation datasets. It would be ideal to validate the identified CpG-exon correlations in independent patient cohorts. However, we were unable to find a multi-omics cancer dataset with exon-level gene expression data, CpG methylation data, and survival data. Therefore, our ability to perform independent validation was limited by data availability. Another limitation pertained to the literature search. Although a comprehensive literature review is a powerful tool for validating and evaluating our results based on existing knowledge, the large number of search results generated by simple searches with keywords made it difficult for us to distinguish useful information efficiently. In addition, experimental results on methylation analysis were rarely reported using CpG id in Tables 1 and 2 as identifiers. Therefore, it is likely that we did not fully capture the relevant literature associated with the correlations observed in this study.

Despite these limitations, this study revealed the relationship between CpG methylation and alternative splicing in cancer and contributed toward the understanding of the role of methylation in alternative splicing during transcriptional processes and carcinogenesis. In addition, survival-related CpG sites not only provided positive indications of the functional relevance of CpGs that correlate with exons, but also served as potential biomarkers predictive of clinical outcomes. In summary, the comprehensive survey of associations between methylation and alternative splicing will facilitate the exploration of the role of methylation regulation in transcriptional processes in cancers.

## Methods

### Data access

Methylation, exon expression, isoform expression, and gene expression data were downloaded from TCGA Genomic Data Commons (GDC) using the GDC Data Transfer Tool. The methylation data used in this study were acquired by the Illumina HumanMethylation450K array, which integrates 485,577 CpG sites on the Illumina chip. Exon, isoform, and gene expression data were measured using the IlluminaHiSeq technology, which included 239,322 exons, 198,619 isoforms, and 23,548 genes, respectively. In addition, this analysis covered 33 different cancer types and data of more than 11,000 cancer patients available in TCGA. We also download the clinical data from GDC, which provided survival outcome data for 11,082 patients.

### Data preprocessing

The gene expression data downloaded from TCGA were normalized by FPKM-UQ [22], and we subsequently transformed the expression data by log-transformation. For each gene, we used StepMiner [16] to compute a global threshold based on all patients across all cancer types. We first sorted the expression data of a given feature for all patients and then fitted a step function to minimize the square error between the original and the fitted values. Since this threshold is derived based on the data of all patients across all cancer types, it is able to robustly define high and low expression. The threshold of each gene was used to binarize the data, so that the patients could be divided into two groups (highly-expressed group vs. lowly-expressed group) according to the binarized expression data of an individual gene.

### Correlation analysis

Correlation analysis was performed using Pearson’s correlation with a Bonferroni correction to the *p*-values based on the number of correlations computed for each cancer type. A correlation analysis was performed between DNA methylation data and expression data of either exons or isoforms, with an FDR-corrected *p*-value threshold of 0.05. All statistical tests were performed using standard Python functions.

### Survival analysis

For each gene and each cancer type, we performed survival analysis on all the individual CpG sites associated with that gene based on the methylation data of patients who highly expressed the gene. Log-rank test was used to assess the statistical significance of the survival difference between patients with the CpG site methylated or unmethylated. *P*-values of the log-rank test were adjusted for multiple testing using Benjamini-Hochberg method with a false-discovery rate (FDR) <0.05. Kaplan-Meier analysis and log-rank tests were performed using the R package “survival”.

### Literature search

A literature search was performed using PubMed, accessed via the National Library of Medicine PubMed interface (http://www.ncbi.nlm.nih.gov/pubmed). We programmatically searched the PubMed database using custom Python scripts. We searched through PubMed for all keywords in all fileds, including the title, abstract and main texts of the articles.

## Supplementary Information

**Additional file 1.** Multi-cancer CpG-exon correlations. This file contains 36,470 CpG-exon correlations across all the cancer types, for CpG-exon pairs that show significant correlations in more than one cancer type.

## Data Availability

All data used in this analysis can be found at the GDC data portal (https://portal.gdc.cancer.gov/).
